# Role of Transient Receptor Potential Canonical Channel 6 (TRPC6) in Diabetic Kidney Disease by Regulating Podocyte Actin Cytoskeleton Rearrangement

**DOI:** 10.1155/2020/6897390

**Published:** 2020-01-03

**Authors:** Qian Wang, Xuefei Tian, Yuyang Wang, Yan Wang, Jialin Li, Tingting Zhao, Ping Li

**Affiliations:** ^1^Beijing Key Laboratory for Immune-Mediated Inflammatory Diseases, Institute of Clinical Medical Sciences, China-Japan Friendship Hospital, Beijing 100029, China; ^2^Beijing University of Chinese Medicine, Beijing 100029, China; ^3^Section of Nephrology, Department of Internal Medicine, Yale University School of Medicine, New Haven, CT 06510, USA; ^4^Department of Nephrology, Guang'anmen Hospital of China Academy of Traditional Chinese Medical Sciences, Beijing 100053, China; ^5^Beijing Key Laboratory of Diabetes Research and Care, Center for Endocrine Metabolism and Immune Diseases, Luhe Hospital, Capital Medical University, Beijing 101149, China

## Abstract

Podocyte injury is an important pathogenesis step causing proteinuric kidney diseases such as diabetic kidney disease (DKD). Actin cytoskeleton rearrangement in podocyte induced by multiple pathogenic factors is believed to be the key process resulting in glomerular injury. Many studies have recently shown that transient receptor potential canonical channel 6 (TRPC6) in podocyte plays a critical role in the development and progression of proteinuric kidney disease by regulating its actin cytoskeleton rearrangement. This review is aimed at summarizing the role of TRPC6 on DKD by regulating the podocyte actin cytoskeleton rearrangement, thereby help further broaden our views and understanding on the mechanism of DKD and provide a theoretic basis for exploring new therapeutic targets for DKD patients.

## 1. Introduction

Transient receptor potential canonical channel 6 (TRPC6) is a nonselective Ca^2+^ channel protein. In 2005, the gene mutation of TRPC6 in podocyte was firstly reported in the patients with focal segmental glomerulosclerosis (FSGS), suggesting the potential importance of TRPC6-mediated Ca^2+^ dynamics for podocyte function [1]. The abnormal expression of TRPC6 in podocyte is closely related to the occurrence of proteinuria in a variety of kidney diseases. It is known that TRPC6 can participate in podocyte injury by regulating the rearrangement of actin cytoskeleton and eventually leads to proteinuria. Podocytes are one of the important components for keeping glomerular filtration barrier integrity and maintenance of its size selectivity. The changes of podocyte number and/or structure of foot process have been demonstrated to be the main cause leading to glomerular proteinuria [2]. The highly organized actin backbone is the molecular basis for the normal structure of podocytes, and it is critical for the maintenance of their morphology and function. In recent years, numerous studies on podocyte have shown that actin cytoskeleton rearrangement induced by multiple pathogenic factors is the key alteration leading to podocyte injury [3, 4], and its mechanisms have remained elusive. Diabetic kidney disease (DKD), one of the most common and severe complication of diabetes mellitus characterized by proteinuria, is the most important cause of end-stage renal disease (ESRD) throughout the world [5]. Podocyte injury is a typical manifestation and a core event in the progression of DKD [6]. More and more studies have shown that aberrant changes of TRPC6 in podocyte play an important role in the proteinuria development and DKD progression. The mechanism may involve the rearrangement of podocyte actin cytoskeleton. However, the precise mechanism has not yet been determined, and further research is urgently needed.

## 2. The Role of TRPCs in Kidney Disease

The transient receptor potential (TRP) superfamily, that are involved in ion homeostasis and/or signal transduction, consists of six subfamilies: transient receptor potential cation channels (TRPCs), melastatin-related TRP proteins (TRPM), vanilloid-receptor-related TRP proteins (TRPV), the ankyrin transmembrane protein (TRPA), mucolipin (TRPML), and polycystin (TRPP) [7–9]. TRPCs are expressed in various segments of the human nephron; they are Ca^2+^-permeant cation channels that depolarize cells and increase intracellular calcium levels. There is a common consensus in TRPC proteins that these channels are potentiated by tyrosine kinase receptor-mediated activation of phospholipase C (PLC) or G protein-coupled receptors (GPCRs). Subsequently, phosphatidylinositol 4,5-bisphosphate (PIP2) is cleaved, liberating diacylglycerol (DAG) and inositol 1,4,5-trisphosphate (IP3) [7, 10]. There are seven different members of TRPCs identified in mammalian cells (TRPC1 through TRPC7) that are subdivided into four subgroups on the basis of protein sequence and function: TRPC1, TRPC2, TRPC4/5, and TRPC3, 6, 7. Each subunit of TRPCs structurally consists of six transmembrane regions, and a pore-forming region is present between the fifth and sixth transmembrane regions. The COOH and NH2-terminals of each TRPC protein subunits localize in the cytosolic portions of the cell [11]. The role of TRPCs in the pathogenesis of various renal disorders is summarized as below (Table 1).

The relationship between TRPC6 and nephropathy has been extensively investigated. TRPC6 mRNA was originally isolated from the brain of mice, and it was also identified in pulmonary artery smooth muscle cells (PASMCs) [22]. TRPC6 is confirmed to be a nonselective cation channel. A large body of studies [23, 24] strongly support that TRPC6 is an essential functional component of TRPCs in vascular smooth muscle cells. It has been shown that activated TRPC6 regulates its contractile function by permeating cation influx and depolarization of vascular smooth muscle cells [25]. Under the regulation of vasoactive hormones and autocrine/paracrine factors [4, 26], podocytes rearrange the F-actin, myosin, and *α*-actinin in the foot processes that change the size selectivity of the glomerular filtration barrier. The size-selectivity property of the glomerular filtration barrier by podocytes is fine-regulated by Ca^2+^ signals [27]. Therefore, TRPC6-mediated calcium influx can be directly associated with actin cytoskeleton rearrangement in podocytes that plays an important role in the proteinuria formation by affecting the intact of glomerular filtration barrier. The finding of FSGS caused by podocyte TRPC6 mutation has shown the proline at amino acid 112 in the TRPC6 protein is substituted with glutamine; it further confirms the importance of TRPC6 and potentially opens up a new view to explore the mechanism of podocyte injury in proteinuric kidney diseases [1].

TRPC6 is mainly expressed in the podocyte slit diaphragm (SD) and adjacent cell membrane. It responds to the activation of various GPCR cascades, resulting in Ca^2+^ influx into podocytes, which played an important role in regulating Ca^2+^ homeostasis in podocyte [21]. The canonical activation pathway for TRPC6 channels was established in 1999 by which certain DAG produced during GPCRs mediated transduction cascades; it evoked an increase in the open probability (OP) of cell surface TRPC6 channels [28]. Membrane-permeable DAG analogs such as 1-oleoyl-sn-glycerol (OAG) increase TRPC6-mediated cationic currents in podocytes, which can be prevented by the inhibition of protein kinase C (PKC) [29]. It is likely that modulation by GPCRs entails two distinct processes, mobilization of TRPC6 channels to the cell surface, and an accompanied increase in the OP of TRPC6 channels already at the cell surface [30]. The gain-of-function (GOF) mutation of TRPC6 in podocyte leads to FSGS and massive proteinuria [1]. It is caused by the impairment of the Ca^2+^-dependent inactivation, resulting from the disruptions of TRPC6's coiled-coil assembly. The excessive Ca^2+^ may contribute to structural damage in the podocytes [31]. Systematic analysis of TRPC6-related mutations showed that loss-of-function (LOF) of TRPC6 has the potential to induce disease [32], which may cause FSGS earlier than in GOF-type TRPC6 patients by interacting with actin cytoskeleton rearrangement during podocyte development [33]. Proteinuria in DKD is also closely related to the abnormal expression of TRPC6 in podocyte [34], and its mechanism is the main research hotspot at present [35].

## 3. TRPC6 Is Associated with DKD Podocyte Injury

TRPC6 channels in normal podocytes remain relatively stable until various stimuli trigger their activation [36]. Although the mechanism for TRPC6 activation is still quite unclear, abnormal changes of angiotensin II (Ang II), reactive oxygen species (ROS), and insulin in DKD setting can stimulate the overexpression of TRPC6 in podocytes. It causes a large amount of Ca^2+^ influx into podocyte, results in foot process effacement, podocyte loss, and other damages, that eventually leads to proteinuria development [36].

The levels of Ang II are highly increased in DKD glomerulus [37]. Interestingly, highly activated podocytes TRPC6 levels can be found in the Ang II-induced renal injury models [38]. Accordingly, using a type 1 DKD animal model induced by an injection of streptozotocin in Dahl salt-sensitive rats (STZ-SS), Ilatovskaya et al. [39] confirmed that Ang II could cause podocyte injury by Ca^2+^ influx through TRPC6, which were consistent with several other studies [34, 40, 41]. ROS plays an important role in the pathogenesis of DKD [42]. ROS, especially hydrogen peroxide forming from NADPH sources by various stimuli, could activate the podocyte TRPC6 in DKD [43]. NADPH oxidases such as Nox2 [44], Nox4 [43], and Nox5 [45] have been shown to be involved in the activation of TRPC6. The abnormalities of insulin signal transduction and pathway have been shown to play the pivotal role on DKD development and progression, as evident by the finding that specific deletion of the gene encoding the insulin receptor causes a loss of podocyte foot processes [46]. Studies have shown that insulin increases the TRPC6 expression on the surface of MPC-5 mouse podocyte cell lines, which is mediated by the NADPH oxidase-dependent ROS production [47]. Podocyte membrane protein urokinase and plasminogen activator receptor (uPAR) and its circulating form (suPAR) play an important role in the development of DKD as well. There were significantly increased uPAR and suPAR levels that were observed in glomeruli and sera of DKD patients, respectively [48], and suPAR could activate TRPC6 via the Nox2-dependent pathway [44]. Additionally, the direct damage of albumin to podocytes can increase the expression of TRPC6 as well [49]. Using an *in vitro* cell model of albumin overload, Chen et al. [49] confirmed that plasma albumin from proteinuric kidney disease can dramatically upregulate the expression of TRPC6 and Ca^2+^ influx in podocytes, followed by podocyte injury. In conclusion, TRPC6 overexpression has been observed in DKD setting that plays a critical role leading to podocyte injury.

## 4. The Association between TRPC6 and Podocyte Actin Cytoskeleton Rearrangement in DKD

### 4.1. Actin Cytoskeleton Rearrangement Participates in DKD Podocyte Injury

#### 4.1.1. Actin Cytoskeleton System and Its Rearrangement

The actin cytoskeleton system is the basic structural and functional unit that regulates cell morphology, cell adhesion, and motility. Different cytoskeletons are distributed in the cell bodies, primary processes, and secondary foot processes of podocytes [5]. As the main functional part of podocytes, foot processes are microfilaments composed of actin molecule especially the F-form actin (F-actin) [50]. A variety of podocyte cytoskeleton-associated proteins interact with F-actin to maintain normal podocyte actin cytoskeletal structure. Three groups of the podocyte cytoskeletal proteins are identified based on their upstream and downstream sequences of actin-influencing pathways: the first group is the actin-binding proteins (ABP) that bind directly to actin by controlling its polymerization and depolymerization; these proteins include *α*-actinin, palladin, synaptopodin, three small guanosine triphosphatase (GTPase) of the Rho family (Rho A, Rac1, and Cdc42), and podocyte-associated talins. The second group is the upstream proteins of the actin-binding proteins; these proteins change the microfilament by regulating actin-binding protein not directly regulating actin; these proteins include SD proteins (nephrin, podocin, and CD2-related proteins) and focal adhesion-associated proteins (integrins, integrin-linked kinase, and focal adhesion kinase). The third group is a larger number of upstream proteins, including TRPCs, multiple kinases, proteases, and circulating permeability factors. They affect the microfilaments and microtubule structures of podocytes by regulating actin through certain signaling pathways. These proteins altogether regulate the polymerization, maintenance, and depolymerization of the actin cytoskeleton by different signaling pathways. The actin cytoskeleton proteins have two major functions depending their localization in the cell: one is the longitudinal actin microfilaments that provide structural support for cells with certain contractile and diastolic functions and also serve as a bridge linking intracellular molecular signaling pathways; the other one is a meshwork of actin filaments existing under the cell membrane that mediate transmembrane proteins and transmembrane signaling pathways [51]. The podocyte injury induced by many pathological factors causes the rearrangement of actin cytoskeleton, which disorganized the original structure of actin fibers, characterized as less-branched and shortened stalks. It resulted in increased mobility, decreased adhesion, increased cell width, foot process effacement, and even a large number of podocytes detachment. Consequently, the function of the glomerular filtration barrier is damaged which facilitates the development of proteinuria. Therefore, regulation or stabilization of the podocyte actin cytoskeleton structure is essential for maintaining normal glomerular filtration function.

#### 4.1.2. Actin Cytoskeleton Rearrangement Results in Podocytes Injury in DKD

More and more evidences have shown that a decrease in podocyte number or density is the strongest predictor of DKD progression; it is positively correlated to the severity of proteinuria [52, 53]. Since podocytes are terminally differentiated epithelial cells and cannot undergo normal cell division [54], the occurrence of a large number of podocyte loss usually indicates that this kind of damage is irreversible. The mechanism by which the changes of podocyte numbers have not been clearly clarified, although apoptosis and active podocyte shedding are indicated the major causes. The apoptosis of podocytes is rarely observed using current experimental techniques available *in vivo*; the damage to podocyte mainly focuses on foot process effacement, podocyte hypertrophy, and podocyte loss; in addition, podocytes collected from the urine of a DKD rat model were cultured in a normal medium for 24 hours; their adherent growth and secreted specific proteins such as podocin were observed [55]. It indicates some podocytes in the urine still have cell activity under the disease state, and the cause of such podocyte reduction and podocyte urine may be the detachment of living podocytes from the glomerular basement membrane (GBM). However, the proportion of podocyte apoptosis and detachment in the mechanism of podocyte loss is still unknown; it can be suggested that the loss of active podocytes from the GBM is indeed an important mechanism for podocyte loss in DKD. As such, the key step of DKD podocyte loss may be attributed to the decreased ability of podocyte adhesion caused by various stimulating factors. It was also confirmed by two-photon microscopy in living zebrafish that the podocyte structure is relatively stable under normal conditions [56]. Dynamic podocyte movement could be observed while it was injured induced by unilateral ureteral ligation and adriamycin administration [57]; signals impairing the focal adhesion proteins can cause podocyte migration. The adhesion ability of podocyte is determined by the cell-matrix adhesion core structure complexes, including integrins/talins/actin, that allow the podocytes to attach to the GBM tightly to maintain the intact of glomerular filtration barrier. Any deficit in the structure components can impair the podocyte adhesion ability [50]. A variety of adhesion proteins existing in focal adhesion complexes, including *α*3*β*1 integrin, integrin-linked kinase, and talins, have all been shown to play a key role in podocyte development and function [58]. Integrin is the most widely studied adhesion protein till now. *β*1 containing integrins are highly expressed in podocytes and are essential for maintaining the structural integrity of the glomerulus [59]. It connects the GBM with the actin skeleton in podocyte cytoplasm through the focal adhesion complexes consisting of talins, vinculin, paxillin, and so on. *α*3*β*1 integrin has been found to be the major integrin form expressing on the surface of the podocyte foot process [60]. The extracellular domain of integrin *α*3*β*1 binds to laminin and fibronectin on GBM, and the intracellular cytoplasmic domain of integrin subunit *β*1 binds to the focal adhesion complexes. Through this specific structure, *α*3*β*1 integrin can regulate the podocyte actin cytoskeleton rearrangement by “outside-in” and “inside-out” signaling pathways that alter the morphology and biological function of podocyte. The abnormal expression or activity of these adhesion proteins affect the function of integrins by changing the adhesion and diffusion of podocytes, regulating the rearrangement of actin cytoskeleton; it finally results in podocyte detachment from GBM. Studies have shown that the expression of *α*3*β*1 integrin is markedly reduced in both DKD patients and high glucose-induced podocyte injury rat models [61]. Additionally, the expression of *α*3*β*1 integrin mRNA and protein in the kidney tissue of DKD rats induced by STZ was significantly decreased [62]. The decreased *β*1 integrin may be accompanied by increased podocyte movement and reduced adhesion [63], and the rupture and stratification of GBM, that eventually caused the loss of podocytes. Recent studies have demonstrated that talin1, a 270 kDa focal adhesion molecule, is important for maintaining podocyte's cytoskeletal stability, podocyte adhesion, and normal renal function [58]. Talin1 is required for integrin subunit *β*1 activation by binding its cytoplasmic domain; any change on talin1 will lead to integrin dysfunction. Talin1 has been recently shown to be associated with the TRPC6-mediated Ca^2+^-dependent calcineurin signal transduction pathway [64] that shed light on the further understanding of TRPC6 on the podocyte biological function.

Foot process effacement is the most important ultrastructural change following podocyte injury. Excluding some hereditary kidney disease, foot process effacement is thought to help damaged podocytes that remain attached to GBM by enhancing the contraction of actin bundles, reducing the risk of podocyte shedding and limiting blood protein leakage into urine; it is an adaptive and reversible reaction [65]. However, the protective effect of this mechanism is limited. As podocytes fall off gradually, the bare GBM area increases accordingly; in the meantime, the abnormal connection of adjacent podocytes caused by the foot process effacement destroys the function of the original SD structure. All of these alternations eventually lead to proteinuria development. Actin cytoskeleton plays a key role in the changes of the foot process because of its high dynamic structure. Disorganized cytoskeletal rearrangement in podocyte causes extension, fusion, and contraction of the foot process [66, 67], followed by SD changes and proteinuria formation. F-actin in podocyte is often observed by an immunofluorescent technique stained with phalloidin. It has been demonstrated that there exist significantly aberrant F-actin pattern changes in DKD. The F-actin pattern alters from more than 90% of cell area filled with thick fibers or at least 2 thick fibers running under the nucleus, into no thick fibers or no fibers visible in the central area of the cell [58, 68–70]. However, the association between the specific modification of this stress fibers and the foot process effacement *in vivo* still requires to be further investigated. In the past decade, the Rho GTPase family proteins have been recognized as an important regulator of proteinuria development [71]; it was also observed in the DKD [72]. Among them, Rho A activation can lead to the fusion of foot processes and the reduction of actin-related proteins. Although there are many reports of the clinical applications for Rho kinase inhibitors on DKD improvement, the exact mechanism remains unclear. Activation of TRPC6 increases the Rho GTPase function and alters the contractile phenotype or stress fibers of the podocyte cytoskeleton by modulating the activity of Rho A and Rac1, resulting in foot process fusion.

### 4.2. TRPC6-Mediated Calcium Influx Leads to DKD Podocyte Actin Cytoskeleton Rearrangement

As the importance of mechanism for actin cytoskeleton rearrangement in podocyte injury and proteinuria formation has been increasingly raised, Ca^2+^ signal transduction and homeostasis disorders in cells have been paid more and more attention that are considered as early events of podocyte injury [73]. Increased intracellular Ca^2+^ concentration in podocytes can be detected in many pathophysiological conditions, such as complement C5b-9 complex-mediated podocyte injury [74] and Ang II-induced podocyte injury [75]. Podocyte injury induced by protamine sulfate (PS) infusion into rat kidney, characterized by foot process effacement and SD fracture, is a classic transit podocyte injury animal model studying the foot process dynamics [76]. Studies on the mechanism of PS-induced podocyte injury have revealed an increased Ca^2+^ concentrations in both cultured podocyte [77] and isolated rat glomeruli [78]. Accordingly, a Ca^2+^-mediated pathway connects those injury factors to podocyte cytoskeleton rearrangement. TRPC6 has been identified to play a major role in the Ca^2+^ homeostasis of podocytes. Therefore, it is speculated that the intracellular Ca^2+^ level of podocytes regulated by TRPC6 plays a key role in maintaining the stability of actin cytoskeleton structure and podocyte biological function.

In order to clarify the consequences of TRPC6 dysregulation, it is important to understand the pathways for the downstream of its activation, both in normal conditions and in abnormal conditions. These are not fully understood and will clearly depend on the cell type examined. At present, the more recognized regulatory mechanism mainly includes two aspects. Firstly, TRPC6 interacts with actin cytoskeleton by the Ca^2+^-dependent Rho GTPase signaling; secondly, TRPC6 acts on podocyte adhesion proteins through the Ca^2+^-dependent calcineurin signal transduction pathway.

#### 4.2.1. TRPC6 Interacts with Actin Cytoskeleton by the Ca^2+^-Dependent Rho GTPase Signaling

The role of Rho GTPase family proteins in the regulation of podocyte actin cytoskeleton has become a research hotspot in recent years [6]. The Rho GTPase family proteins, including Rho A, Rac1, and Cdc42, are major regulators of cytoskeletal dynamics [79]. Rho A can promote the formation of cell body and terminal actin-myosin stress fibers to regulate the stability of podocyte cytoskeleton; Rac1 and Cdc42 can regulate the formation of filopodium and lamellipodium, respectively, further promoting an increase of podocyte movement [80]. In a DKD mouse model, it has been observed an unbalanced activation of Rho A, Rac1, and Cdc42; these changes result in a rearrangement of the podocyte actin cytoskeleton and following foot process effacement. Either upregulation or inhibition of Rho A expression in podocytes can cause foot process effacement [81]; increased activity of Rac1 and Cdc42 may result in a significant decrease in F-actin expression [69, 82]. Studies have shown that the mechanism by which Cdc42 alters cell migration may be through regulation of the transcription factor serum response factor (SRF) to regulate *β*1 integrin expression at a transcriptional level [83]. While *β*1 integrin is one of the important molecules for the linkage between cell with extracellular matrix. This is a functional change in the Rho GTPase signaling pathway that alters cell migration by *β*1 integrin. Either loss of cdc42, or loss of SRF, or loss of *β*1 integrin in podocytes can lead to proteinuria and progressive kidney failure. SLIT-Robo *ρ*GTPase-activating protein 2a (SRGAP2a) belongs to the Rho GTPase family as a member of the SRGAPs family. Recent studies have shown [84] that SRGAP2a expression in podocytes from DKD patients and *db/db* mice, an established animal model of type 2 diabetes, was significantly reduced compared to normal control. Overexpression of SRGAP2a could reverse previously abnormal actin cytoskeletal rearrangement, inhibit podocyte migration, and alleviate foot process effacement by inhibiting the overactivated Rho A and Cdc42.

Ca^2+^ influx induced by TRPC6 channel activation can increase Rho A activity and further increase intracellular Ca^2+^ concentration by combining with Rho-associated coiled-coil protein kinase (ROCK), resulting in disorganized F-actin arrangement and foot processes effacement in podocytes [85]. Tian et al. [86] found that TRPC6 activated by Ang II bound to Rho A which mediated the stress fiber contraction phenotype of podocytes; TRPC5 was coupled with Rac1 which mediated the migration phenotype of podocytes. These two channels antagonized each other to regulate podocyte cytoskeletons. It was suggested that the imbalance of Rho A and Rac1 mediated by TRPC6 and TRPC5 could be one of the main causes leading to podocyte injury. However, the TRPC5 function described above has been challenged by some recent studies. Overexpression of TRPC5 in mice does not show proteinuria nor does it aggravate proteinuria induced by lipopolysaccharide (LPS) [19]. On the contrary, the loss of podocyte stress fibers was observed in the study of podocyte TRPC6 overexpression [21]. Recent study has established conditionally immortalized TRPC6 knockout (KO) podocytes isolated from TRPC6 KO mice [87]. Compared with wild-type (WT) TRPC6 podocytes, they have less cell motility, greater adhesion, and more actin stress fibers. This difference may be attributed to the difference in TRPC6 knockout level caused by siRNA technology used in previous studies, where some preservation of expression is seen. Heparan sulfate proteoglycan syndecan-4 (Sdc4) can interact with TRPC6, promotes its expression, and transports it to the cell surface in podocytes [88], resulting in reduced activation of Rho A and increased activation of Rac1, accompanied by increased generation of ROS and total *β*3-integrin [89]. All these results suggest that TRPC6 plays a more direct and critical role in podocyte actin cytoskeleton rearrangement.

Insulin can cause foot process effacement through controlling contraction of podocyte actin [46], which is mediated by TRPC6-dependent Ca^2+^ influx [90]. Recent studies have shown that TRPC6-dependent activation of the adenosine 5′-monophosphate-activated protein kinase (AMPK) signaling pathway is a novel mechanism, by which insulin mediates actin cytoskeletal rearrangement of podocyte. Phosphorylation of AMPK*α*2 induced by TRPC6 is required for activation of the insulin-dependent Rac1 signaling pathway in podocytes [91]. This study further elucidates that Rac1 may regulate F-actin rearrangement in podocytes by phosphorylation of actin regulatory proteins cofilin and P21 protein-activated kinase 1 (PAK1). Additionally, insulin can disrupt the interaction between microfilaments by activating protein kinase G type I*α* (PKGI*α*) subunits of rat podocytes, causing the podocyte actin cytoskeleton to rearrange [92]. Subsequent studies have confirmed that TRPC6 mediates the above pathway and considers insulin-dependent rearrangement of the cytoskeleton to be TRPC6 and PKGI*α*-dependent [90].

#### 4.2.2. TRPC6 Acts on Podocyte Adhesion Proteins by the Ca^2+^-Dependent Calcineurin Signal Transduction Pathway

Talins are one of the crucial adhesion proteins present in focal adhesion complexes. Talins bind and activate integrins, coupling it to the actin cytoskeleton [93]. There are two isoforms of talin in vertebrates: talin1 and talin2. Talin1 is widely expressed in all cytoplasm and can be cleaved by calpain, a calcium-dependent nonlysosomal cysteine protease [94]. It can control the affinity of actin binding through methyltransferase Ezh2 [95], playing an important role in mediating cell adhesion [96] and early embryonic development [97]. Talin2 is present at a higher level in the cerebral cortex, heart muscle, and kidneys. Both talin1 and talin2 are expressed in podocyte. Podocyte-specific deletion of talin1 presented the progressive proteinuria, foot process effacement, and kidney failure. Ablation of podocyte talin2 in mice lacking talin1 did not significantly change its phenotype, suggesting that talin1 function most likely predominates in normal podocyte physiology [58]. As one of the most important activated molecule on integrins, the change of talin1 can lead to integrins dysfunction, which is associated with the emergence of a variety of pathological phenomena including thrombosis, stroke, and cancer metastasis [98]. The recent study explores the importance of talin1 in the field of kidney disease for the first time [58] and demonstrated that mice lacking talin1 specifically in podocytes display severe proteinuria, foot process effacement, and renal failure. Although the lack of talin1 only caused a mild decrease in *β*1 integrin activation and podocyte adhesion, significant actin cytoskeleton rearrangement was observed in the isolated primary podocytes.

**Figure 1:**
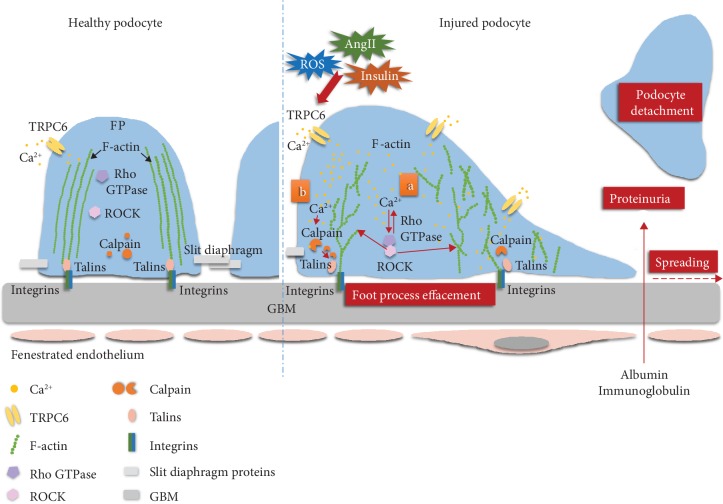
TRPC6 is involved in the rearrangement of podocyte actin cytoskeleton. In DKD, various stimulating factors lead to high expression of TRPC6 and a large amount of Ca^2+^ influx, causing podocyte hypertrophy and foot process effacement. By binding Rho GTPase family proteins to ROCK, F-actin becomes disordered, shortened, and branched (a), or talins are abnormally cleaved by activation of calpain (b), causes the destruction of podocyte focal adhesion complex structure, increased motility, and ultimately leads to foot processes effacement, detachment of podocytes, and proteinuria. Abbreviations: FP: foot process; TRPC6: transient receptor potential canonical channel 6; Rho GTPase: Rho guanosine triphosphatase (Rho A, Rac1, and Cdc42); ROCK: Rho-associated coiled-coil protein kinase; GBM: glomerular basement membrane; ROS: reactive oxygen species; Ang II: angiotensin II.

Calpain is a family of nonlysosomal cysteine proteases that are mainly activated by Ca^2+^; its overactivity is closely related to renal injury [99]. Calpain can induce calcineurin activation. Activated calcineurin is involved in proteinuria development by cleaving the podocyte actin cytoskeletal proteins. Calcium/calcineurin signaling pathway is speculated to play a crucial role in regulating the stability of podocyte cytoskeletal structure. The calcineurin inhibitor cyclosporin A (CsA) is one of the current mainstream treatments on proteinuric kidney diseases through its immunosuppressant role; meanwhile, the CsA's protective role mediating by stabilizing podocyte actin cytoskeleton has been paid more and more attention as well. The signaling pathway of calcium/calcineurin is initiated by the movement of Ca^2+^ from the extracellular space into cells that mediates through TRPC6. After entry into the cells, Ca^2+^ binds to calcineurin and activates its phosphatase activity. Activated calcineurin dephosphorylates cytoplasmic nuclear factor of activated T-cell (NFATC) family members including NFATC1 through NFATC4, resulting in their translocation to the nucleus, where they function as transcription factors to regulate target gene expression. The downstream signaling pathway cascade of TRPC6 involves a Ca^2+^-dependent calcineurin signal transduction, which usually activates NFATCs. The important transcriptional target of NFATCs is TRPC6 itself that results in a harmful feedforward loop. However, this activation pathway has not been examined in podocytes. Accordingly, Verheijden et al. [64] examined whether the calcium-dependent protease calpain1 mediates TRPC6-dependent podocyte injury in human and experimental FSGS, as well as in cultured podocytes. They found that TRPC6-dependent Ca^2+^ influx increases the activity of calpain1 and leads to podocyte injury through loss of talin1 and activation of calcineurin. Their study highlights that TRPC6-dependent activation of calpain1 could be a key mechanism on the initiation and progression of podocyte injury in glomerular diseases. Overall, the authors suggest that both TRPC6 and calpain1 could be the future therapeutic targets in the treatment of proteinuria and prevention of glomerular injury in which they are overactivated. In addition, TRPC6 can activate calpain directly through the extracellular signal-regulated kinase (ERK1/2) pathway in a Ca^2+^-independent manner [100], thereby regulating cytoskeleton rearrangement, adhesion, and motility ability of podocyte [87] (Figure 1).

How do the TRPC6/calpain change in the DKD podocyte injury? Our results showed that there was a significantly loss of the podocyte talin1 on the mRNA level and protein level in a DKD mouse model (unpublished), which was strongly positively correlated to the severity of proteinuria and podocyte actin cytoskeleton rearrangement. Undoubtedly, further investigation on the podocyte actin cytoskeletal changes is very important in elucidating podocyte biological function, which will broaden our view and deepen our understanding on its role in the proteinuric kidney disease. In addition to the above-mentioned proteins, there are still many other cytoskeleton proteins that can participate in DKD podocyte injury through TRPC6-mediated calcium influx. For example, a recent study has found reduced MYH9 in DKD patients, type 2 DKD mouse model, and Ang II-stimulated podocytes [100]. MYH9 is one of the heavy chain isoforms of nonmuscle myosin II (NM II) involved in cell migration, adhesion, and cellular actin cytoskeleton contraction [101]. It indicates that the decrease of MYH9 expression mediated by Ang II in DKD can induce F-actin rearrangement in podocytes and subsequent proteinuria, which may be related to TRPC6-dependent Ca^2+^ influx due to NOX4-mediated ROS production.

## 5. Conclusions

In conclusion, TRPC6 plays a crucial role in podocyte injury in proteinuric kidney diseases and it participates in proteinuria formation by regulating its actin cytoskeleton rearrangement. Although the investigation on the role and mechanism of TRPC6 in DKD podocyte actin cytoskeleton rearrangement is still in its infancy, these results all suggest that it is of great value to study the mechanism of DKD podocyte injury targeting TRPC6 that provide a potential therapeutic target on TRPC6 for DKD. It is noteworthy that type 1 DKD model Akita mice lacking TRPC6 have only a short-term renal protective effect by inhibiting albuminuria and reducing tubular injury, while mesangial dilatation and insulin resistance subsequently aggravate renal injury, suggesting that LOF-type mutation of TRPC6 has the same pathogenic effect in type 1 DKD [102]. Most studies available focus on the experiments from type 1 DKD animal models *in vivo* and podocyte culture *in vitro*; the researches on human DKD and type 2 DKD animal models are limited. Therefore, how to accurately clarify the role of TRPC6 in the development of DKD, especially in type 2 DKD, and elucidate the specific regulatory mechanism of TRPC6 on actin cytoskeleton rearrangement of DKD podocytes are the key points for further investigation.

## Figures and Tables

**Table 1:** Overview of renal expression, function, and associated disorders of TRPCs.

TRPCs	Location of expression in kidney	Role in kidney	Kidney diseases or injuries associated with TRPC disorders	Objects of research	References
TRPC1	MC; PTEC; TAL	Regulation of mesangial cell contractility; regulation of Ca^2+^ influx	Diabetic nephropathy	Human; ZDF rats; STZ-induced rats; Ang II-induced human MC	[12, 13]
TRPC2	A pseudogene and does not form functional channels in human				[14]
TRPC3	P; CDEC; RF	Regulation of Ca^2+^ influx	Autosomal dominant polycystic kidney disease; renal interstitial fibrosis	TRPC3^−/−^ mice; UUO rats; CiPTEC cells with siTRPC3; IMCD3 cells treated with pcDNA/TRPC3; RF treated by the pyrazole-derivative TRPC3 blocker pyr3	[15, 16]
TRPC4	MC	Store-operated Ca^2+^ entry	Unknown	Mouse MC line (CRL-1927) under high-glucose conditions	[17]
TRPC5	P	Regulation of Ca^2+^ influx; regulation of actin cytoskeleton rearrangement (need to be further investigated)	Progressive kidney diseases Focal segmental glomerulosclerosis	TRPC5-KO mice by LPS injection; mouse podocytes treated by PS, LPS, and Cch	[18, 19]
TRPC6	P; MC EC; CDEC	Regulation of Ca^2+^ influx; regulation of actin cytoskeleton rearrangement	Focal segmental glomerulosclerosis; minimal change disease; membranous glomerulonephritis; type-1 diabetes; renal fibrosis	Human; PHN rats; C5b-9-attacked podocytes; HEK293 cells recombined with mouse GFP-TRPC6	[20, 21]
TRPC7	Unknown				

Abbreviations: MC: mesangial cell; PTEC: proximal tubule epithelial cells; TAL: thick ascending limb of the loop of Henle; P: podocytes; CDEC: collecting duct epithelial cells; RF: renal fibroblasts; EC: endothelial cells; ZDF: Zucker diabetic fatty; STZ: streptozotocin; Ang II: angiotensin II; CiPTEC: human conditionally immortalized proximal tubular epithelial cells; IMCD3: mouse inner medullary collecting duct cell line; UUO: unilateral ureteral obstruction; LPS: lipopolysaccharide; PS: protamine sulfate; Cch: carbachol; PHN: the passive Heymann nephritis (PHN) model; HEK: human embryonic kidney.
